# Marsupialization before enucleation as a treatment strategy for a large calcifying odontogenic cyst: Case report

**DOI:** 10.1016/j.ijscr.2020.01.031

**Published:** 2020-01-27

**Authors:** Antonia Taiane Lopes de Moraes, Haroldo Arid Soares, João de Jesus Viana Pinheiro, André Luís Ribeiro Ribeiro

**Affiliations:** a School of Dentistry, Federal University of Pará (UFPA), Belém, PA, Brazil; b Oral Diagnostic Service, Doctor Carmino Caricchio Municipal Hospital, São Paulo, Brazil

**Keywords:** Calcifying odontogenic cyst, Marsupialization, Odontogenic cysts, Oral pathology

## Abstract

**Introduction:**

Odontogenic pathological lesions can generally be divided into cysts and tumors. Calcifying odontogenic cyst (COC) is a non-aggressive odontogenic cyst, however, in a few occasions, COC can show excessive growth and destruction. This study describes an uncommon aggressive presentation of a large COC and discusses the treatment strategies used to achieve a favorable result.

**Presentation of case:**

A 11-year-old child sought treatment for a painless growth on his mandible that caused facial asymmetry. On clinical and imaging examination, a large radiolucent lesion was observed in a panoramic x-ray involving the right side of the mandible. Radiopaque areas were observed within the lesion. Treatment comprised of an initial marsupialization followed by surgical removal of the lesion. Follow up extended for more than 10 years and no signs of recurrence were observed.

**Discussion:**

Initial marsupialization was successfully employed in order to decrease the lesion, inducing bone formation, and reducing the risk of mandibular fracture and inferior alveolar nerve damage. Four months after marsupialization, the lesion substantially reduced its volume and enucleation with peripheral ostectomy was carried out. This approach allowed a safer surgical removal of the lesion and achieve an excellent result.

**Conclusion:**

Although some authors consider marsupialization as an unnecessary step that delays final treatment, when well indicated, it facilitates surgical treatment, promotes and preserves normal tissues, reduces the necessity of surgical reconstruction and costs, yet showing excellent results.

## Highlights

• Two-stage treatment strategies are effective for large lesions.
• Marsupialisation promotes lesion decompression and decreases lesion volume.
• Reduction of the lesion allows lower risk enucleation of the structures.
• This strategy prevents damage to important anatomical structures.

## 1 Introduction

Calcifying odontogenic cysts (COC), first described in 1964 by Gorlin et al. [1], are benign odontogenic lesions, which originate from the odontogenic epithelium and are part of a spectrum of lesions characterized by calcification of “ghost cells” of the odontogenic epithelium [2]. Although COCs may occur in soft tissue, they are most commonly found as intraosseous lesions [3]. Average age is around 33 years and most cases are diagnosed between the 2nd and 4th decades of life [4]. There is no gender predisposition, and intraosseous and extraosseous forms occur proportionally in the maxilla and mandible; however, the region of the incisors and canines are the most affected, with 65% of cases [3]. Curettage is the most common treatment method [5].

Odontogenic lesions can achieve large dimensions [6], because of this, surgeons sometimes opt for conservative techniques, avoiding inconvenient sequels and preserving important anatomical structures of the stomatognathic system [7]. One of these conservative treatments is decompression/marsupialization followed by enucleation and curettage [8].

Marsupialization was first described by Partsch as a treatment of cystic lesions [9]. It consists of opening the lesion and communicating it to a body cavity, such as the oral cavity or paranasal sinuses. A surgical window is created by suturing the oral mucosa to the lesion wall, thus creating a temporary long communication with the oral cavity. This procedure reduces the internal hydrostatic pressure, allowing free drainage of any liquid that otherwise would accumulate within the lesion. It results in beneficial effects, such as size reduction, bone formation and metaplasia od pathological tissues, all associated with a better prognosis [10].

This study aimed to show the importance of using conservative techniques in the surgical management of a large odontogenic cyst in a young patient. This case demonstrate that performing a few invasive procedures [11] can be a favorable strategy for preventing extensive surgical damage as observed in single-stage surgical treatments.

## 2 Presentation of case

An 11-year-old male patient presented to the oral diagnostic service of our institute, complaining of swelling inside the mouth on the right-hand side of the face, that showed evidence of a slow and asymptomatic growth.

**Fig. 1:**
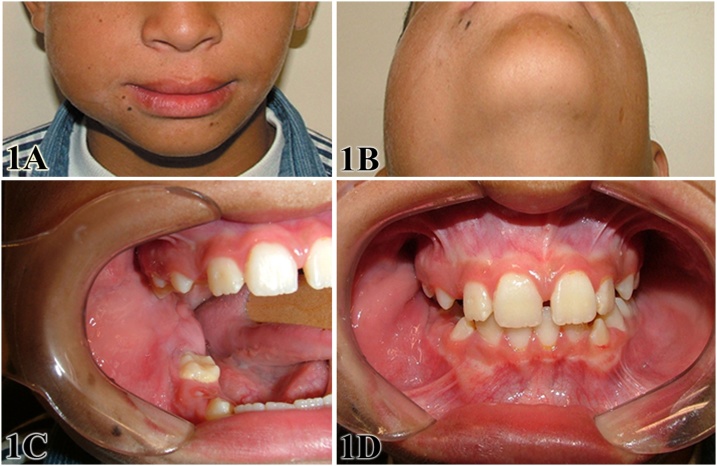
Extraoral aspect showing growth of the right-hand side of the patient’s face resulting in marked facial asymmetry (A and B). Intraoral aspect showing a painless bone growth clearly involving the buccal cortical bone (C and D).

Physical examination showed facial asymmetry due to a painless growth on the right mandibular region (Fig. 1A), which was in the body, angle and ascending ramus of the mandible (Fig. 1B). Intraorally, a painless, firm, mass could be seen covered by and normal looking mucosa, clearly involving the buccal cortex and extending from the region adjacent to the lower first molar to the coronoid process (Fig. 1C). The affected area measured about 7 cm in its largest diameter, reason why was considered a large lesion (Fig. 1D).

**Fig. 2:**
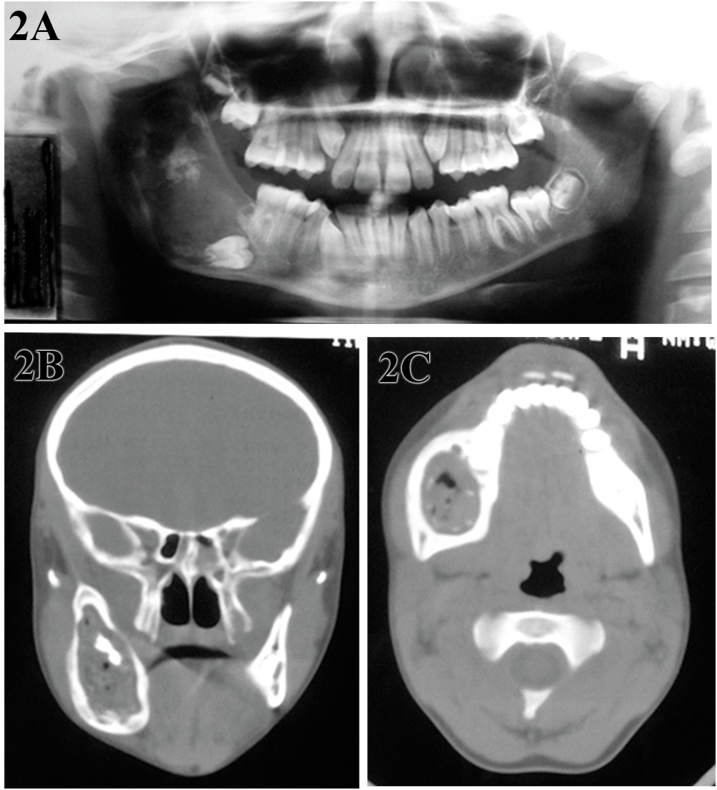
Panoramic radiograph showing a well-defined multilocular radiolucent area in the region from the right lower first molar to the coronoid process, areas of calcification and tooth germ 47 (A). Sagittal (B) and axial (C) computed tomography (CT) cuts showed a well-defined hypodense area, as well as basal cortical bulging of the mandible.

Panoramic radiography revealed a well-defined, homogeneous multilocular radiolucent lesion extending from the right lower first molar region to the coronoid process, with the presence of calcified areas and inclusion of tooth germ 47 (Fig. 2A). On sagittal (Fig. 2B) and axial cuts (Fig. 2C) cuts of computed tomography (CT), a well-defined hypodense area as well as a basal cortical bulging of the mandible.

**Fig. 3:**
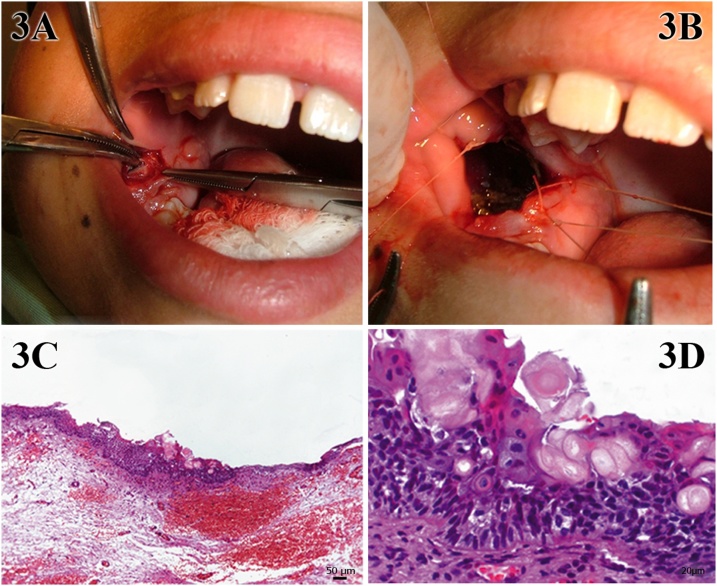
Initial stage of treatment corresponding to marsupialization (A and B). Isles of odontogenic epithelial remains scattered and partially lining the capsule of highly hyalinised dense connective tissue (C), consisting of 3–4 layers of hyperchromatic cells with cubic basal or high columnar layers, and numerous eosinophilic ghost cells (D).

An incisional biopsy (Fig. 3A) was performed under local anesthesia as a step of the marsupialization technique, in which the oral cavity mucosa was sutured with the lesion edge (Fig. 3B). An elliptical fragment of the normal mucosa and lesion capsule was removed and referred for pathological analysis, which revealed islands of scattered odontogenic epithelial and a partial lining of hyaline dense connective tissue capsule (Fig. 3C). The lesion was composed of 3–4 layers of hyperchromatic cells with cubic basal or high columnar layers and numerous eosinophilic ghost cells (Fig. 3D). These findings confirmed the diagnosis of a COC.

**Fig. 4:**
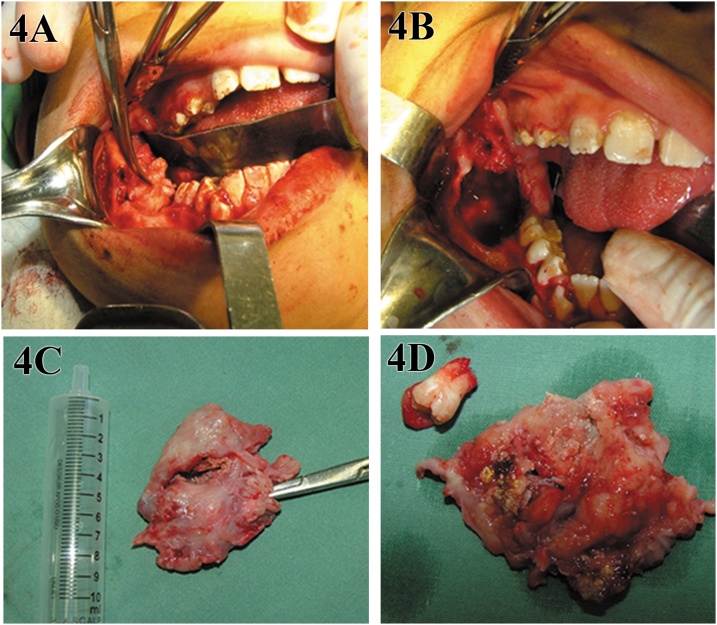
Second stage of treatment, corresponding to total enucleation with peripheral ostectomy (A and B). Tissue removed by surgery (C), including tooth 47 (D).

The patient remained under clinical radiographic follow-up for four months, during that time, a progressive decrease in lesion size and new bone formation were observed. After this period, cyst reduction slowed and changes in lesion size and bone formation have established. At this moment, surgical enucleation with peripheral osteotomy was carried out under general anesthesia (Fig. 4A–C). Tooth 47 was also removed due to its association with the pathological tissues (Fig. 4D). This maintained the integrity of the inferior alveolar nerve and reduced the risk of mandibular fracture.

**Fig. 5:**
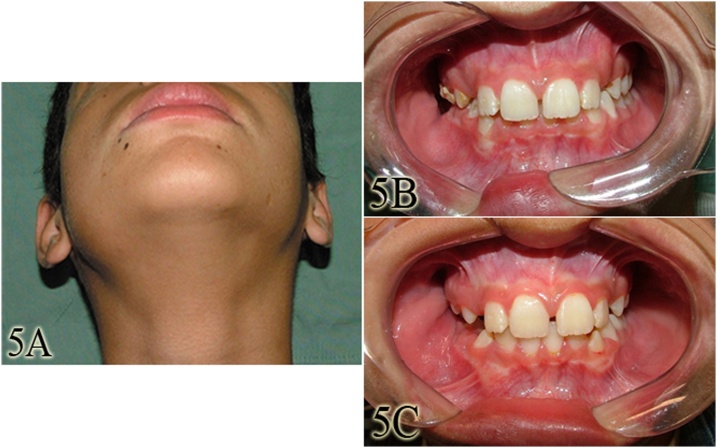
Extraoral aspect after one year of treatment (A). Intraoral aspect, showing significant improvement, with absence of any signs of recurrence of the lesion, as well as preservation of occlusion and mouth opening (B and C).

One year after surgery, the patient had no sensorial deficits or any signs of recurrence of the lesion. There was also a major improvement in facial asymmetry (Fig. 5A), with natural recontour of previously expanded bone. Both occlusion and mouth opening were preserved (Fig. 5B and C) and no functional deficit was observed. The patient was followed-up for more than 10 years and remains with no signs of recurrence. This work has been reported in line with the SCARE criteria [12].

## 3 Discussion

Initially described by Gorlin et al. in 1962 [1], COC is a rare odontogenic lesion, representing <2% of all odontogenic tumors and cysts [13]. Most COCs present as an intraosseous lesion. Since its identification, there has been controversy regarding its terminology and classification. This debate in the literature has resulted in the definition of two histological variants, cystic and neoplastic, a classification of great importance to the surgeon regarding the treatment to be employed [14]. Lesions classified as cystic usually contain a wall of connective tissue lined with odontogenic epithelium, with the presence of ghost cells, with different levels of calcification. To these lesions, a surgical approach such as enucleation and curettage is recommended [15].

COC lesions defined as tumors have a more locally destructive behavior, containing a proliferation of odontogenic epithelial islands, and their connective tissue has varying amounts of dentinoid. As a result, treatment modalities for these lesions involve a more aggressive surgical approach. Depending on the size of the lesion, this approach may be in the form of peripheral ostectomy or segmental resection [16].

The lesion described in this current case had histological, radiographic and clinical characteristics of a cyst variant of COC. The gold standard treatment for COC is enucleation and curettage. However, because it was a large lesion and involved a young patient, a conservative approach using an initial marsupialization followed by total enucleation was chosen [17].

Marsupialization has as its main objective the reduction of lesion size, and therefore, reducing the need for a more extensive and radical surgery [18]. As a result, a more predictable enucleation in terms of three-dimensional regression is possible, resulting in an easier removal of all pathological tissues, thus decreasing the chances of recurrence. In addition, bone remodelling is promoted, as well as osteogenesis [19].

Successful treatment of large odontogenic cysts using initial marsupialization and a second phase surgery with enucleation and curettage has been previously reported [[19], [20], [21]]. When lesions show large dimensions, as seen in the present case, a two-stage approach allows important structures to be preserved. This preservation reduces the sequels of treatment and the needs of aggressive and expensive surgical reconstruction. This treatment modality also allowed the preservation of the inferior alveolar nerve, mandibular contour and normal facial development. It also prevented fracture of the mandible and a decreased the risk of recurrence, providing a better quality of life to the patient [22].

## 4 Conclusion

Marsupialization prior to total enucleation of a large COC proved to be a favorable and effective treatment choice. Since this surgical maneuver promoted a significant reduction of the lesion, damage to anatomical structures was insignificant and bone repair was facilitated. Together, this approach reduces the overall morbidity and costs, doesn´t require surgical reconstruction and results in an excellent outcome.

## Funding

This research did not receive any specific grant from funding agencies in the public, commercial, or not-for-profit sectors.

## Ethical approval

We declare that our institution does not require ethical approval of clinical case reports.

## Consent

Written informed consent was obtained from the patient’s parent for publication of this case report and accompanying images. A copy of the written consent is available for review by the Editor-in-Chief of this journal on request.

## Author contribution

JJVP and ALRR contributed in conceptualisation, ATLM, HAS, contributed in study concept and design, JJVP and ATLM contributed in writing the paper.

## Registration of research studies

None.

## Guarantor

The guarantor of this work, Joao de Jesus Viana Pinheiro, accept full responsibility for the study and the conduct of the study, had access to the data, and controlled the decision to publish.

## Provenance and peer review

Not commissioned, externally peer-reviewed.

## Declaration of Competing Interest

All authors declare no conflict of interest in formulating this article.
